# Primary Pure Squamous Cell Carcinoma of the Duodenum: A Case Report

**DOI:** 10.4021/gr2010.01.1333

**Published:** 2010-01-20

**Authors:** Tadashi Terada

**Affiliations:** Department of Pathology, Shizuoka City Shimizu Hospital, Miyakami 1231 Shimizu-Ku, Shizuoka 424-8636, Japan. Email: piyo0111jp@yahoo.co.jp

**Keywords:** Duodenum, Squamous cell carcinoma, Histopathology

## Abstract

Only two cases of squamous cell carcinoma of the duodenum have been reported in the literature. The author herein reports a case of squamous cell carcinoma of the duodenum. A 75-year-old man was admitted to our hospital because of nausea, vomiting, and weakness. An endoscopic examination revealed a duodenal tumor. The tumor was circumferential elevated one with duodenal obstruction, and located in the descending part near and distal to the ampulla of Vater. Imaging modalities also detected the duodenal tumor, but did not show tumors of other locations including the pancreas. The duodenal tumor was different from ampullary tumor and pancreatic tumor. Six biopsies were obtained from the duodenal tumor. All the six biopsies showed malignant cells arranged in a medullary pattern. The malignant cells showed hyperchromatic nuclei, and mitotic figures were scattered. Keratinization and intercellular bridges were recognized. The pathologic features were interpreted as a squamous cell carcinoma of the duodenum. The carcinoma was pure squamous cell carcinoma without differentiation into adenocarcinoma or endocrine carcinoma. Operation was not possible because of the patient’s age and weakness. The patient was treated by chemotherapy and radiation, but he showed a downhill course. Metastases emerged, and he died of systemic metastasis 17 months after the first presentation. Autopsy was not performed.

## Introduction

Carcinoma of the duodenum is very rare. Most of the duodenal carcinoma is adenocarcinoma [1]. A review of the literature revealed only two cases of squamous cell carcinoma of the duodenum [2, 3]. Adenosquamous carcinoma was also reported [4]. The author encountered a case of primary pure squamous cell carcinoma of the duodenum and reported herein.

## Case Report

A 75-year-old man was admitted to our hospital because of nausea, vomiting, and weakness. An endoscopic examination revealed a duodenal tumor. The tumor was circumferential elevated one with duodenal obstruction, and located in the descending part near and distal to the ampulla of Vater. Imaging modalities also detected the duodenal tumor, but did not show tumors of other locations including the pancreas. The duodenal tumor was different from ampullary tumor and pancreatic tumor. Six biopsies were obtained from the duodenal tumor, and they were diagnosed as squamous cell carcinoma as described below. Operation was not possible because of the patient’s age and weakness. The patient was treated by chemotherapy and radiation, but he showed a downhill course. Metastases emerged, and he died of systemic metastasis 17 months after the first presentation. Autopsy was not performed.

**Figure 1:**
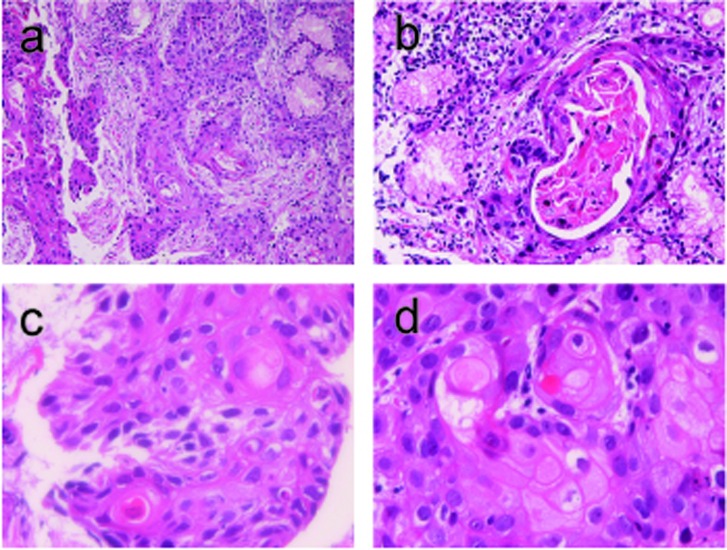
(a) Low power view of duodenal squamous cell carcinoma. HE, x 40. (b) Keratinization is recognized. HE, x 200. (c) Individual keratinization and intercellular bridges are recognized. HE, x 400. (c) Obvious intercellular bridges are recognized. HE, x 400.

All the six biopsies showed malignant cells arranged in a medullary pattern (Fig.1a). The cells showed hyperchromatic nuclei, and mitotic figures were scattered. Keratinization was recognized in several areas (Fig. 1b, c). Intercellular bridges were also recognized in several areas (Fig. 1c, d). The pathologic features were interpreted as a squamous cell carcinoma. The carcinoma was pure squamous cell carcinoma without differentiation into adenocarcinoma or endocrine carcinoma.

## Discussion

Pure squamous cell carcinoma of the duodenum is extremely rare. The present case was a carcinoma with keratinization and intercellular bridges, thus fulfilling the criteria of squamous cell carcinoma. The present carcinoma did not show other differentiations. Therefore, the present case is a pure squamous cell carcinoma.

The present case showed a circumferential elevated tumor with duodenal obstruction in the duodenum. Imaging modalities revealed no tumors in other organs including the pancreas. Endoscopically, the present tumor was located near and distal to the ampulla of Vater. The present case was not ampullary carcinoma and pancreatic carcinoma. Therefore, the primary site of the present tumor was duodenum.

Most of the duodenal carcinoma develops in the second portion near the ampulla [1]. This is because the periampullary sites are irritated by pancreatic juice and bile, putative mitogens. The present tumor was also located near and distal to the ampulla.

The pathogenesis of squamous cell carcinoma of the duodenum is only speculative. Barnhill et al [5] reported an interesting tumor of the duodenum. The tumor showed tripartite differentiations, ie, adenocarcinoma, squamous cell carcinoma, and neuroendocrine carcinoma [5]. He speculated that their case had arisen from duodenal pluripotential stem cells capable of differentiating into multiple cell types [5]. The present case might have arisen from such pluripotential stem cells.

In summary, the author reported an extremely rare case of primary pure squamous cell carcinoma of the duodenum. Such a tumor may arise from pluripotential stem cells in the duodenum.
